# HIV-1 Nef blocks autophagy in human astrocytes

**DOI:** 10.1080/15384101.2015.1105700

**Published:** 2015-12-23

**Authors:** Luca Sardo, Sergey Iordanskiy, Zachary Klase, Fatah Kashanchi

**Affiliations:** 1 Department of Biological Sciences; University of the Sciences; Philadelphia, PA USA; 2 National Center for Biodefense and Infectious Diseases; George Mason University; Manassas, VA USA

HIV is a pandemic virus and the causative agent of AIDS. The development of combination antiretroviral therapy (cART) significantly improved the length and quality of life of HIV infected individuals. Despite the success of cART, 50% of patients develop HIV associated neurocognitive disorders (HAND).^1^ HAND is the result of HIV infection in the central nervous system (CNS), leading to attention deficit, behavior changes and memory impairment.^2^ In the post-cART era, aging HIV patients will likely suffer from HAND, therefore efforts are currently dedicated to understand the mechanisms of HIV induced neurocognitive disorders.

Autophagy is a cellular catabolic process that degrades endogenous and exogenous components. The major histocompatibility complex exposes the degradation products of autophagy to the cellular membrane thereby activating the immune system. Autophagy evolved in response to stress, promoting cell survival during starvation and resistance to pathogens. Autophagy deficiency in cells of the CNS has been ascribed to aging related neurocognitive disorders such as Alzheimer and Parkinson's diseases.^1^ Some pathogens reduce or block autophagy to evade the immune system. Inefficient clearance of pathogen-derived proteins leads to cellular physiological dysfunction causing toxic effects.^3^ Even if the viral replication is controlled by cART, HIV proteins persist in cellular reservoirs. The Negative Regulatory Factor (Nef) and the Trans-Activator of Transcription (Tat) proteins accumulate in cells of the CNS and impair autophagy.

Nef targets the expression of the CD4 receptor, preventing cell superinfection. It is highly expressed, is excreted from cells and is abundant in the serum. The precise mechanism of Nef secretion, whether as free protein or associated with exosomes, is still a subject of debate. However it is clear that Nef can be taken up by bystander cells. For the first time, Saribas et al. investigated the activity of Nef toward autophagy in astrocytes.^4^ Astrocytes support cells of the blood-brain barrier and function as nutrient supply and repair of the CNS. HIV infects a small proportion of astrocytes despite their lack in CD4, and this is sufficient to induce neurocognitive disorders.

The authors transduced primary human fetal astrocytes with an adenoviral vector expressing Nef, and could detect Nef by immunocytochemistry. Nef was abundantly expressed 48 hours after transduction and the levels of LC3-II and p62 autophagy markers were higher than in the untreated control (Ad-Null). The microtubule-associated protein 1A/1B-light chain 3-II (LC3-II) is derived from the cytosolic LC3-I protein by phosphatidylethanolamine conjugation. LC3-II is recruited at the autophagosome membrane, which is fused to a lysosome to form an autophagolysosome within which cellular components, including LC3-II, are hydrolyzed.^5^ The polyubiquitin binding sequestosome 1 protein (p62/SQSTM1) recruits polyubiquinated proteins to the autophagosome. Inhibition of autophagy leads to increasing levels of p62. Other autophagy markers, the autophagocytosis associated protein 3 and 7 (Atg3 and Atg7), which support the conversion from LC3-I to LC3-II, Beclin-1 and the apoptosis regulator BAX were not altered by Nef expression in astrocytes. The article nicely demonstrates that Nef expression mimics the action of Bafilomycin A1 (BafA1), an antibiotic molecule that impedes the fusion of autophagosome to lysosome. BafA1 induces the accumulation of LC3-II and p62 proteins in astrocytes at similar level as Nef. By detecting immunofluorescent LC3-II, the authors confirmed that Nef blocks the autophagy at the autophagosome stage.

In a previous report, astrocytes expressing Nef were implanted in rats' brain and impaired animals' novel location and object recognition. Nef induced the upregulation of the monocytes chemoattractant chemokine ligand 2 (CCL2), infiltration of macrophages in the hippocampus and loss of CA3 neurons.^2^ Another group showed that HIV-1 Nef inhibits the nuclear localization of the Transcription Factor EB (TFEB) inhibiting autophagy in macrophages.^6^ A recent study showed that HIV Tat expression in neuronal cells reduced the autophagosome markers LC3-II and p62/SQSTM1 levels, and is able to partially reverse Baf1 effects. Tat also interacts with the lysosomal associated membrane protein LAMP2, suggesting an intervention of Tat in the autophagosome and lysosome fusion.^7^ There are indications that Nef and Tat differentially regulate autophagy, blocking and promoting its catabolic function.

Human cells counteract HIV infection by degrading viral components. The interplay between HIV proteins and different cell types of the CNS remains unclear. The insurgence of neurocognitive disorders in aging HIV patients requires efforts to elucidate the function of viral proteins in respect to autophagy and to develop strategies for targeting HIV replication.
